# Microalbuminuria: a sentinel of neurocognitive impairment in HIV-infected individuals?

**DOI:** 10.1007/s00415-019-09674-6

**Published:** 2020-01-24

**Authors:** Antoine Moulignier, Anne-Claire Viret-Vilayphon, François-Xavier Lescure, Emmanuelle Plaisier, Laurence Salomon, Cédric Lamirel, Gilles Pialoux, The ALCOVE Study Group

**Affiliations:** 1 Memory Clinic, Department of Neurology, Fondation Adolphe de Rothschild, 29, rue Manin, 75019 Paris, France; 2 Department of Infectious and Tropical Diseases, Assistance Publique-Hôpitaux de Paris, Hôpital Tenon, Sorbonne Universités, UPMC Université Paris 6, Paris, France; 3 Department of Infectious and Tropical Diseases, Assistance Publique-Hôpitaux de Paris, Hôpital Bichat-Claude-Bernard, Université Paris Diderot, IAME, UMR1137, Sorbonne Paris Cité, Paris, France; 4 Department of Nephrology and Dialysis, Assistance Publique-Hôpitaux de Paris, Hôpital Tenon, Sorbonne Universités, UPMC Université Paris 6, INSERM, UMR-S 1155, Paris, France; 5 Department of Clinical Research, Fondation Adolphe de Rothschild, Paris, France; 6 Department of Ophthalmology, Fondation Adolphe de Rothschild, Paris, France

**Keywords:** Cerebral small-vessel disease (CSVD), HIV infection, Microalbuminuria, Neuropsychological assessment, Urine albumin/creatinine ratio (UACR)

## Abstract

**Background:**

According to population-based studies, microalbuminuria is associated with subsequent cognitive decline over a 4–6-year period, because of cerebral small-vessel disease (CSVD). This prospective cross-sectional study (NCT02852772) was designed to evaluate whether a history of microalbuminuria is associated with subsequent cognitive decline in combined antiretroviral therapy (cART)-treated persons living with human immunodeficiency virus (PLHIVs).

**Methods:**

From our computerized medical database, we identified 30 PLHIVs (median age 52 years), immunovirologically controlled on cART, who had microalbuminuria in 2008 and had undergone, between 2013 and 2015, a comprehensive neuropsychological assessment (NPA) including seven domains (cases): information-processing speed, motor skills, executive functions, attention/working memory, learning/memory, reasoning and verbal fluency. Forty-nine PLHIVs matched for age (median age 48 years; *p* = 0.19), sex, and year of first HIV-seropositivity without microalbuminuria in 2008 were identified and underwent the same NPA between 2013 and 2015 (controls).

**Results:**

Cases performed less well than controls for information-processing speed (*p* = 0.01) and motor skills (*p* = 0.02), but no differences were found for the other cognitive domains and global *z*-scores. A multivariable linear-regression model adjusted for confounding factors confirmed the microalbuminuria effect for the information-processing-speed *z* score.

**Conclusion:**

cART-treated PLHIVs with a history of microalbuminuria subsequently had worse cognitive performances for the information-processing-speed domain, possibly because of CSVD. Our observations should be considered preliminary findings of a temporal link between microalbuminuria, CSVD, and subsequent cognitive impairment.

## Introduction

Because the renal and cerebral microcirculations share common hemodynamic properties, characterized by high-flow and low-resistance end organs with tightly autoregulated perfusion, the concept of the kidney being an anatomical and functional brain surrogate is increasingly recognized [1, 2]. Kidney-damage markers are microalbuminuria and/or lower estimated glomerular filtration rate (eGFR) [2]. Magnetic resonance imaging-documented cerebral small-vessel disease (CSVD) and/or neurocognitive impairment could reflect brain damage [2]. Over a decade, a growing body of evidence has shown a significant relationship between those kidney-damage markers and cognitive decline [2–4], leading to the concept of reno-cerebrovascular disease, considered to be based on SVD due to endothelial dysfunction, accompanied by inflammation and oxidative stress [5, 6].

In the general population, microalbuminuria is associated with a vascular profile of cognitive impairment appearing within 4–6 years [7–9]. Independent of traditional vascular risk factors, microalbuminuria is also associated with CSVD, which is responsible for impaired information-processing speed, motor skills, and executive functioning [8–12]. CSVD affects up to 50% of middle-aged persons living with HIV (PLHIVs), despite combined antiretroviral therapy (cART)-controlled aviremia [13]. The increased risk of CSVD is not associated with exposure to any ART class [14]. Evidence supports a significant CSVD role in the development of milder forms of HIV-associated neurocognitive disorders (HAND) for cART-treated virus-suppressed PLHIVs, leading to the new paradigm of vascular-driven milder forms of HAND [15–17].

While a comprehensive neuropsychological assessment (NPA) is required to characterize cognitive functions and diagnose HAND, it is time-consuming, personnel intensive, and costly. Simpler and less expensive approaches may suffice to detect PLHIVs at risk of cognitive decline that will lead to a thorough NPA in this selected PLHIV sub-population.

We hypothesized that PLHIVs with microalbuminuria would have more neurocognitive impairment than those without. Herein, impairment evaluation was limited to three specific cognitive domains—information-processing speed, motor skills, and executive function—known to be sensitive to the more premature, subtle decline of cART-controlled PLHIVs [17–19]. To evaluate, in this preliminary study, microalbuminuria’s role in cognitive function, we selected PLHIVs without severe diabetes mellitus and/or hypertension, hepatitis C virus (HCV) infection, past or ongoing neurological diseases (notably acquired immunodeficiency syndrome (AIDS)-defining neurological events), and/or alcohol or illicit drug addiction.

## Methods

### Study population

The prospective, cross-sectional ALCOVE study (NCT02852772) was designed to estimate the association between microalbuminuria assessed with the urine albumin/creatinine ratio (UACR) 5 years earlier and current cognitive impairment, regardless of suspected cognitive impairment signs or symptoms or subjective cognitive complaints. In 2008, three of us (FX-L, EP, GP) established a cohort to explore HIV-associated kidney disease; 694 PLHIV outpatients were enrolled, among 2750 followed in the Department of Infectious Disease, Tenon Hospital, Paris, France [20]. Among those 694 PLHIVs, 96 had microalbuminuria at inclusion. Between 2013 and 2015, we selected among the 96 microalbuminuric PLHIVs, all those complying with the following inclusion criteria (henceforth cases): ≥ 18 years old; known HIV-positivity for ≥ 5 years; current CD4^+^ T-cell count ≥ 350/µl; cART-controlled plasma-HIV load (plVL) < 40 copies/ml for at least 12 months; and 2008 UACR, assessed on a fresh morning urine sample, 3–30 mg/mmol. Exclusion criteria were: neurological and psychiatric diseases or prior/current neurological and psychiatric pathologies including AIDS-defining neurological events (i.e., stroke, seizure disorders, multiple sclerosis, dementia, traumatic brain injury with loss of consciousness), HCV infection, prior/current alcohol or illicit substance abuse (except for occasional cannabis or popper use, < 1/month), diabetes mellitus with microvascular complications, uncontrolled hypertension, absence of fasting glycemia and lipidemia testing for ≥ 1 year, HIV-associated nephropathy, insufficient command of French and/or eGFR < 15 ml/min/1.73 m^2^. Two non-microalbuminuric PLHIVs from the same database were matched to each case for age (± 5 years), sex, and year of first HIV-seropositivity (± 5 years), with 2008 UACR assessed on a fresh morning urine sample, < 3 mg/mmol (henceforth controls).

Entry sociodemographic characteristics, lifestyle (tobacco, alcohol and drugs), blood pressure, HIV/AIDS history, other past/current neurological conditions, psychotropic drug use, cardiovascular risk factors, and all treatments were recorded.

This study was approved by the CPP Île-de-France VI, Groupe Hospitalier Pitié-Salpêtrière Ethics Committee, and adhered to the tenets of the Declaration of Helsinki. Written informed consent was obtained from all participants.

### Neuropsychological assessment

Trained neuropsychologists, blinded to UACR, administered a comprehensive NPA covering seven cognitive domains (information-processing speed, motor skills, executive functions, attention/working memory, learning/memory, reasoning and verbal fluency). The main stumbling block of the Frascati criteria [21] is that they label > 30% of a normative reference population as cognitively impaired, which yields an unreasonably high false-positive proportion of cognitively impaired participants in a study population, due to their lower-than-expected specificity [19, 22–25]. Because evidence is mounting in support of not using the HAND criteria in the modern cART era in resource-rich settings and the availability of updated standards, we chose to apply the validated, normative datasets threshold of impairment, as widely acknowledged in memory clinics [26]. Raw scores for each cognitive domain test, except the 9-hole peg test, were normalized to age, sex, and educational levels, yielding *z* scores for analyses. In addition, a specific multivariable-model analysis with age, sex, and educational level was run for the 9-hole peg test to compensate for its lack of a *z* score. Within-domain test scores were averaged to calculate domain-specific *z* scores and across domains to calculate a global *z* score. Hamilton Depression and Anxiety Rating Scales assessed depression and anxiety, and Cognitive Complaint Interview evaluated subjective cognitive complaints [27, 28].

### Statistical analyses

Non-parametric Mann–Whitney *U* tests for continuous variables, expressed as median [interquartile range] and Fisher’s exact tests for categorical variables, expressed as number (%), compared cases versus controls. Based on those comparisons of characteristics, multivariable linear-regression models using the NPA scores as dependent variables were adjusted for variables that differed between them. Variables achieving *p* ≤ 0.10 in non-parametric analyses were included in the multivariable regression model. CDC stage C and CD4^+^ T-cell nadir, two of the main variables associated with HAND in cART-treated aviremic PLHIVs, were also added to this model. Statistical analyses were computed with Statistica software (Statsoft, Inc, Maison Alfort, France).

### Data-availability statement

Anonymized data will be shared on request by any qualified investigator provided that data transfer is in agreement with EU legislation on general data-protection regulations.

## Results

Between November 2013 and January 2015, 31 cases and 60 controls were recruited. Nine patients dropped out, did not reply or missed their scheduled NPAs. Three PLHIVs were excluded secondarily because of unreliable NPA (language difficulties), leaving 30 cases and 49 controls whose characteristics are reported in Table 1. Cases had lower anxiety scores, current CD4^+^ T-cell counts, males having sex with males, and smoking rates than controls but were comparable for all other variables. Cases had significant cognitive impairment compared to controls for information-processing speed (*p* = 0.01) and motor skills (*p* = 0.02), but their other domains and global *z* scores were comparable. After Bonferroni correction for the three comparisons defined as our primary objective, only the information-processing-speed difference remained significant.

**Table 1:**
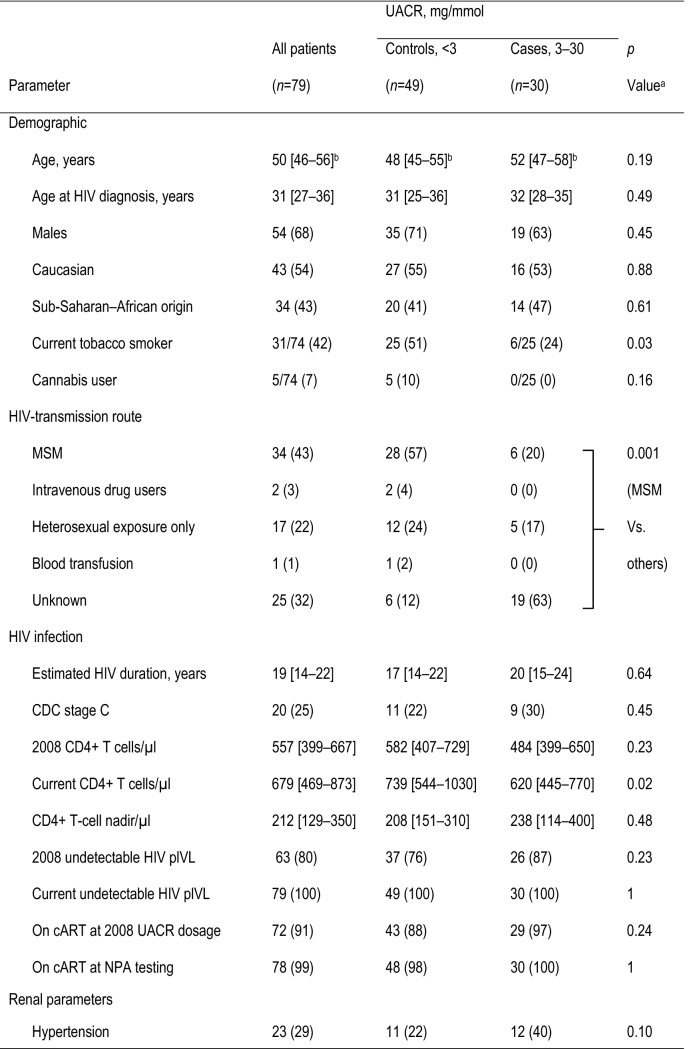

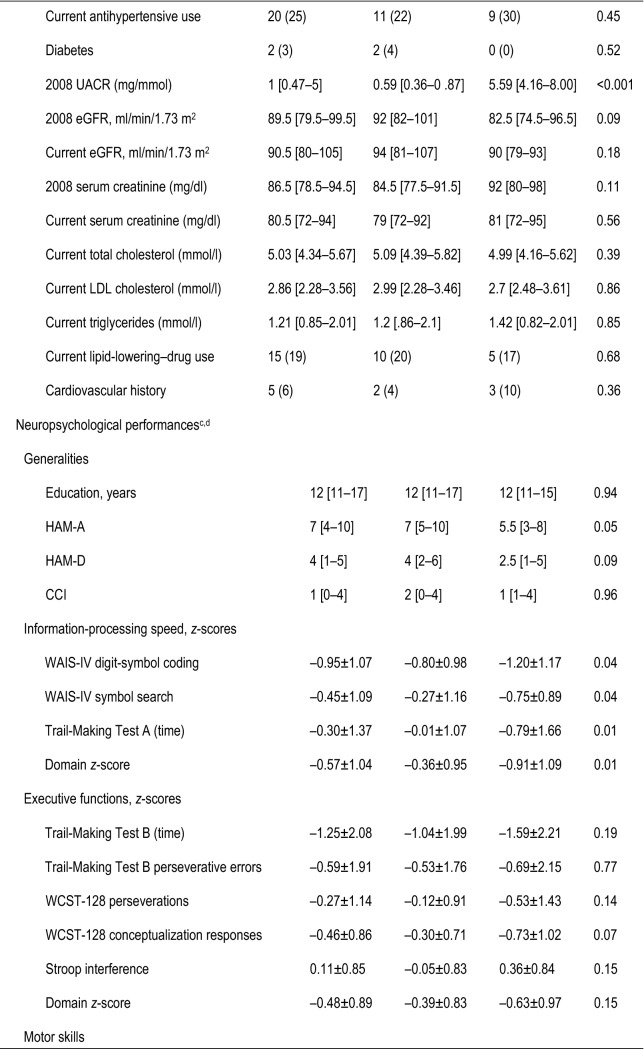

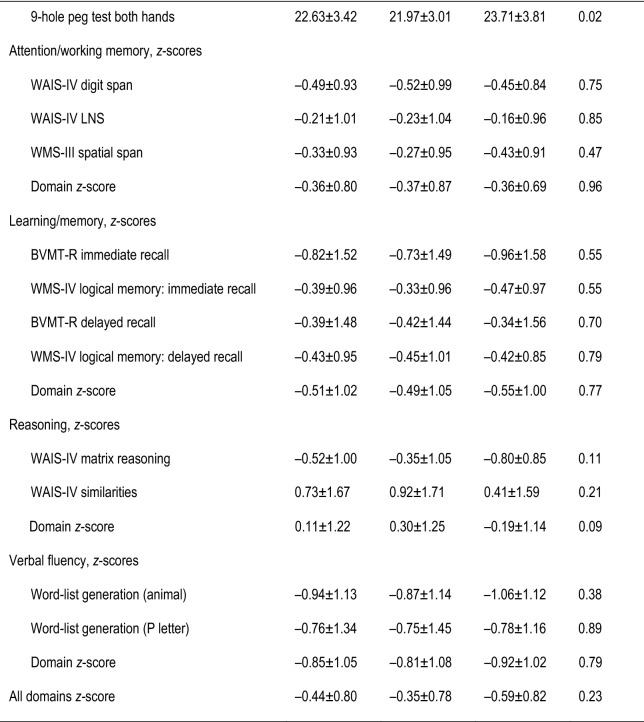
Epidemiological, clinical and biological characteristics and neuropsychological assessment (NPA) performances of 79 HIV-infected patients

^a^Significantly different values are in bold type

^b^Values are median [interquartile range] or number (%)

^c^All test results are demographically corrected *z* scores, except the 9-hole peg test

^d^Values are expressed as median [interquartile range] or mean ± SD

*BVMT-R* Brief Visuospatial Memory Test-Revised, *cART* combined antiretroviral treatment, *CCI* Cognitive Complaint Interview, *CDC* Centers for Diseases Control, *eGFR* estimated glomerular filtration rate, *HAM-A* Hamilton Anxiety Rating Scale, *HAM-D* Hamilton Depression-Rating Scale, *LDL* low-density lipoprotein, *LNS* Letter-Number Sequencing, *MSM* males having sex with males, *PASAT* paced auditory serial addition, *plVL* plasma virus load, *Stroop* color and word test, *UACR* urine albumin/creatinine ratio, *WAIS-IV* Weschler Adult Intelligence Scale-4th edition, *WCST* Wisconsin Card-Sorting Test, *WMS-IV* Weschler Memory Scale 4th edition, *WMS-III* Weschler Memory Scale 3rd edition

To confirm the microalbuminuria effect on information-processing speed and motor skills, we used multivariable linear-regression models with information-processing-speed *z* score and 9-hole peg test both hands score as dependent variables. These multivariable models were adjusted for the following variables: current tobacco smoker, hypertension, microalbuminuria, Centers for Diseases Control (CDC) stage C, 2008 eGFR, CD4^+^ T-cell nadir, and the current CD4^+^ T-cell count (Table 2). Because it was unknown for 63% of the cases, HIV-transmission route appeared less useful and was not retained. Presence of microalbuminuria was retained as being associated with lower information-processing-speed *z* scores but was no longer associated with poorer performance in the 9-hole peg test, even after including age, sex, and educational level for the latter, to compensate for its lack of a *z* score. CDC stage C was also associated with lower information-processing-speed *z* scores.

**Table 2:** Multivariable analyses

Variables	*z* score information-processing speed		9-hole peg test both hands	
	*β* (95% CI)^a^	*p* value^b^	*β* (95% CI)^a^	*p* value^b^
Current tobacco smoker	0.19 (− 0.04;0.43)	0.11	− 0.00 (− 0.26;0.26)	0.98
Presence of hypertension	0.05 (− 0.18;0.27)	0.69	− 0.12 (− 0.37;0.13)	0.34
Presence of microalbuminuria	− **0.32 (**− **0.57;0.06)**	**0.01**	0.03 (− 0.25;0.31)	0.84
CDC stage C	− **0.26 (**− **0.50;0.04)**	**0.02**	− 0.17 (− 0.43;0.13)	0.18
2008 eGFR	− 0.20 (− 0.42;0.02)	0.07	− 0.09 (− 0.34;0.16)	0.47
Current CD4^+^ T-cell count	− 0.23 (− 0.48;0.01)	0.06	0.07 (− 0.21;0.34)	0.63
CD4^+^ T-cell count nadir	− 0.03 (− 0.27;0.20)	0.77	− 0.01 (− 0.27;0.26)	0.95

*95% CI* 95% confidence interval, *CDC* Centers for Diseases Control, *eGFR* estimated glomerular filtration rate

^a^*β* statistic values represent the standardized coefficients in multiple linear-regression models

^b^Significant *p* values are in bold type

## Discussion

We applied the exposed/non-exposed study design [29, 30], as was used for the general population [7], to our historical cohort of cART-treated PLHIVs, whose inclusion/exclusion criteria have been described elsewhere [20]. Indeed, it appeared to be the most cost-effective way to test, in a preliminary study, whether microalbuminuria detected 5 years earlier was significantly associated with PLHIVs’ subsequent cognitive impairment. After adjustment for factors associated with HAND and/or microalbuminuria (i.e., age, educational level, hypertension, CD4^+^ T-cell nadir), previously microalbuminuric cases had worse cognitive performances for the information-processing-speed domain than controls. Overall, our PLHIVs performed well in executive-functioning and memory domains, which agrees with recent studies on cART-controlled aviremic PLHIVs without severe comorbidities [18, 19, 22].

A UACR (≥ 22.6 mg/mmol)–HAND association was reported [31]. However, that study was hampered by an unusual definition of albuminuria, predominantly cART-naïve PLHIVs, with 40% immunovirologically uncontrolled individuals (plVL ≥ 200 copies/ml), who had only recently started cART, with potential factors contributing to HAND (e.g., current cocaine use and HCV coinfection) and insufficient NPA results and CD4 nadir. UACR ≥ 3 mg/mmol was shown to be a determinant of poorer cognitive performance by middle-aged, cART-controlled, aviremic PLHIVs compared to HIV-uninfected individuals [17].

Systemic SVD is considered the underlying pathophysiological mechanism, with renal SVD manifesting as microalbuminuria and brain involvement as cognitive decline, with hemodynamic similarities in the vascular beds of both end organs [1, 2]. CSVD, which affects 50% of PLHIVs [13] and is a major contributor to HAND in multivariable analyses, seems even more important than HIV parameters [17]. Information-processing speed is one of the most affected domains in the general population with CSVD-related cognitive decline [32, 33] and PLHIVs with primary HAND [34]. Information-processing speed being the only deficiency found in our cART-controlled-aviremic cases might suggest CSVD [32]. Unfortunately, our study design did not include brain magnetic resonance imaging to explore the microalbuminuria–CSVD relationship, representing a clear limitation. However, our objective was not to show an association between microalbuminuria and CSVD in PLHIVs, and such imaging is technically demanding, costly and time-consuming, unlike inexpensive, safe, easy, and rapid microalbuminuria testing, especially relevant for low-resource countries.

Although UACR measurements without cognitive testing preceded the NPA by up to 5 years, our study should be considered cross-sectional. As for similar studies [35], our historical PLHIV cohort did not undergo cognitive testing at the time they were screened for microalbuminuria. A longitudinal study is needed to evaluate the temporal link between microalbuminuria, CSVD, and subsequent cognitive impairment. Also, our study-sample size was relatively small, so the absence of difference in other cognitive domains might be due to a lack of power. However, information-processing speed was the most affected domain, as attested by the two major tests used to assess it, i.e., Trail-Making Test B and Wechsler Memory Scale 4th edition, while language, memory and motor skill functions were preserved [36].

Our cohort is representative of PLHIVs in Northern Europe, where > 90% are successfully treated [17], but our findings may not be generalizable to more vulnerable PLHIVs. Nevertheless, the 2019 UNAIDS world epidemiological data showed that 79% of PLHIVs are aware of their seropositivity, 78% of PLHIVs knowing their HIV status are cART-treated, and 86% of those cART-treated PLHIVs have a plVL below the detection threshold (unaids.org). Moreover, it was recently demonstrated that low detectability-threshold plVLs of 51–200 or 201–500 copies/ml were strongly associated with virological failure [37]. Hence, reporting results nowadays concerning virologically uncontrolled cART-treated PLHIVs is not really suitable. UACR, measured with a single-spot urine sample, correlated well with 24-h urinary albumin-excretion rates [38, 39].

## Conclusion

Our observations should be considered preliminary to devising well-designed, prospective, longitudinal studies to confirm the temporal microalbuminuria–CSVD link with subsequent cognitive impairment. Indeed, microalbuminuria is a rapid inexpensive test, of particular interest in low-resources countries. Because the PLHIV CSVD rate is twice that of the general population, HIV infection could serve as a model to evaluate the microalbuminuria–cognitive decline relationship in the general population.

## References

[1] Lee M, Ovbiagele B (2011). Reno-cerebrovascular disease: linking the nephron and neuron. Expert Rev Neurother.

[2] Mogi M, Horiuchi M (2011). Clinical interaction between brain and kidney in small vessel disease. Cardiol Res Pract.

[3] Toyoda K (2015). Cerebral small vessel disease and chronic kidney disease. J Stroke.

[4] Chillon J-M, Massy ZA, Stengel B (2016). Neurological complications in chronic kidney disease patients. Nephrol Dial Transplant.

[5] Monk RD, Bennett DA (2006). Reno-cerebrovascular disease? The incognito kidney in cognition and stroke. Neurology.

[6] Jabbari B, Vaziri ND (2018). The nature, consequences, and management of neurological disorders in chronic kidney disease: neurology and chronic kidney disease. Hemodial Int.

[7] Georgakis MK, Dimitriou NG, Karalexi MA (2017). Albuminuria in association with cognitive function and dementia: a systematic review and meta-analysis. J Am Geriatr Soc.

[8] Georgakis MK, Chatzopoulou D, Tsivgoulis G, Petridou ET (2018). Albuminuria and cerebral small vessel disease: a systematic review and meta-analysis. J Am Geriatr Soc.

[9] Weiner DE, Bartolomei K, Scott T (2009). Albuminuria, cognitive functioning, and white matter hyperintensities in homebound elders. Am J Kidney Dis.

[10] Martens RJH, Kooman JP, Stehouwer CDA (2017). Estimated GFR, albuminuria, and cognitive performance: the Maastricht study. Am J Kidney Dis.

[11] Wada M, Nagasawa H, Kurita K (2007). Microalbuminuria is a risk factor for cerebral small vessel disease in community-based elderly subjects. J Neurol Sci.

[12] Weiner DE, Gaussoin SA, Nord J (2017). Cognitive function and kidney disease: baseline data from the systolic blood pressure intervention trial (SPRINT). Am J Kidney Dis.

[13] Moulignier A, Savatovsky J, Assoumou L (2018). Silent cerebral small-vessel disease is twice as prevalent in middle-aged individuals with well-controlled, combination antiretroviral therapy-treated human immunodeficiency virus (HIV) than in HIV-uninfected individuals. Clin Infect Dis.

[14] Januel E, Godin O, Moulignier A (2019). Impact of ART classes on the increasing risk of cerebral small-vessel disease in middle-aged, well-controlled, cART-treated, HIV-infected individuals; Microvascular Brain Retina And Kidney (MicroBREAK) Study Group. J Acquir Immune Defic Syndr.

[15] Cysique LA, Brew BJ (2019) Vascular cognitive impairment and HIV-associated neurocognitive disorder: a new paradigm. J Neurovirol **(Epub ahead of print)** 10.1007/s13365-018-0706-5 30635846

[16] Su T, Wit FWNM, Caan MWA (2016). White matter hyperintensities in relation to cognition in HIV-infected men with sustained suppressed viral load on combination antiretroviral therapy. AIDS.

[17] Schouten J, Su T, Wit FW (2016). Determinants of reduced cognitive performance in HIV-1-infected middle-aged men on combination antiretroviral therapy. AIDS.

[18] Janssen MAM, Meulenbroek O, Steens SCA (2015). Cognitive functioning, wellbeing and brain correlates in HIV-1 infected patients on long-term combination antiretroviral therapy. AIDS.

[19] Meyer A-CL, Boscardin WJ, Kwasa JK, Price RW (2013). Is it time to rethink how neuropsychological tests are used to diagnose mild forms of HIV-associated neurocognitive disorders? Impact of false-positive rates on prevalence and power. Neuroepidemiology.

[20] Lescure F-X, Fellahi S, Pialoux G, et al (2019) Prevalence of tubulopathy and association with renal function loss in HIV-infected patients. Nephrol Dial Transplant **(Epub ahead of print)** 10.1093/ndt/gfz081 31071216

[21] Antinori A, Arendt G, Becker JT (2007). Updated research nosology for HIV-associated neurocognitive disorders. Neurology.

[22] McDonnell J, Haddow L, Daskalopoulou M (2014). Minimal cognitive impairment in UK HIV-positive men who have sex with men: effect of case definitions and comparison with the general population and HIV-negative men. J Acquir Immune Defic Syndr.

[23] Underwood J, De Francesco D, Leech R (2018). Medicalising normality? Using a simulated dataset to assess the performance of different diagnostic criteria of HIV-associated cognitive impairment. PLoS ONE.

[24] Gisslén M, Price RW, Nilsson S (2011). The definition of HIV-associated neurocognitive disorders: are we overestimating the real prevalence?. BMC Infect Dis.

[25] Nightingale S, Winston A, Letendre S (2014). Controversies in HIV-associated neurocognitive disorders. Lancet Neurol.

[26] Azam B, Whitfield TJ, Radford D (2016). Trends in referred patient profiles in a memory clinic over 20 years. Dementia.

[27] Hamilton M (1967). Development of a rating scale for primary depressive illness. Br J Soc Clin Psychol.

[28] Thomas-Antérion C, Honoré-Masson S, Laurent B (2006). The cognitive complaint interview (CCI). Psychogeriatrics.

[29] Röhrig B, du Prel J-B, Wachtlin D, Blettner M (2009). Types of study in medical research: part 3 of a series on evaluation of scientific publications. Dtsch Arzteblatt Int.

[30] Omair A (2016). Selecting the appropriate study design: case–control and cohort study designs. J Health Spec.

[31] Kalayjian RC, Wu K, Evans S (2014). Proteinuria is associated with neurocognitive impairment in antiretroviral therapy treated HIV-infected individuals. J Acquir Immune Defic Syndr.

[32] Prins ND, van Dijk EJ, den Heijer T (2005). Cerebral small-vessel disease and decline in information processing speed, executive function and memory. Brain.

[33] ter Telgte A, van Leijsen EMC, Wiegertjes K (2018). Cerebral small vessel disease: from a focal to a global perspective. Nat Rev Neurol.

[34] Fellows RP, Byrd DA, Morgello S (2014). Effects of information processing speed on learning, memory, and executive functioning in people living with HIV/AIDS. J Clin Exp Neuropsychol.

[35] Sacre JW, Magliano DJ, Zimmet PZ (2018). Associations of chronic kidney disease markers with cognitive function: a 12-year follow-up study. J Alzheimers Dis.

[36] Makinson A, Dubois J, Eymard-Duvernay S, et al (2019) Increased prevalence of neurocognitive impairment in aging people living with human immunodeficiency virus: the ANRS EP58 HAND 55–70 Study. Clin Infect Dis **(Epub ahead of print)** 10.1093/cid/ciz670 31755936

[37] Fleming J, Mathews WC, Rutstein RM (2019). Low level viremia and virologic failure in persons with HIV infection treated with antiretroviral therapy. AIDS.

[38] Dyer AR (2004). Evaluation of measures of urinary albumin excretion in epidemiologic studies. Am J Epidemiol.

[39] Bakker AJ (1999). Detection of microalbuminuria. Receiver operating characteristic curve analysis favors albumin-to-creatinine ratio over albumin concentration. Diabetes Care.

